# Integrative systems biology identifies SIRT1 as a senescence-related theranostic target in atherosclerosis and poliumoside as a natural activator

**DOI:** 10.3389/fphar.2026.1805938

**Published:** 2026-05-22

**Authors:** Xiaolong Wang, Runlu Shi, Chen Ji, Huilin Li, Ziwen Jia, Xin Tian, Xi Xie, Yanping Wang, Qiao-Ping Wang

**Affiliations:** 1 Laboratory of Metabolism and Aging, School of Pharmaceutical Sciences (Shenzhen), Sun Yat-sen University, Shenzhen, Guangdong, China; 2 Department of Pharmacy, Ningxia Chinese Medicine Research Center, Yinchuan, Ningxia, China; 3 State Key Laboratory of Optoelectronic Materials and Technologies, Guangdong Province Key Laboratory of Display Material and Technology, School of Electronics and Information Technology, Sun Yat-sen University, Guangzhou, China; 4 Guangdong Provincial Key Laboratory of Diabetology, The Third Affiliated Hospital of Sun Yat-sen University, Guangzhou, China; 5 State Key Laboratory of Anti-Infective Drug Discovery and Development, School of Pharmaceutical Sciences, Sun Yat-sen University, Guangzhou, China

**Keywords:** atherosclerosis, bioinformatics, cellular senescence, natural products, poliumoside, SIRT1

## Abstract

**Background:**

Cellular senescence contributes to atherosclerotic plaque progression and instability, yet senescence-related diagnostic biomarkers and actionable targets remain incompletely defined.

**Purpose:**

To identify senescence-related diagnostic/therapeutic targets in atherosclerosis using integrative systems biology, and to prioritize natural products that modulate the lead target with experimental validation.

**Methods:**

Transcriptomic profiles from GSE21545 (126 carotid plaques and 97 paired peripheral blood mononuclear cell samples) were analyzed to identify differentially expressed genes and intersected with CellAge senescence genes. WGCNA and two machine-learning approaches (LASSO and SVM-RFE) were used for feature selection and target prioritization, followed by ROC analysis, functional enrichment, immune infiltration analysis, and PPI network construction. A natural product library (5,565 compounds) was screened against SIRT1 via hierarchical docking, MM-GBSA rescoring, and 100-ns molecular dynamics simulations. Lead compound activity was tested in H_2_O_2_-injured HUVECs using CCK-8 assays, qRT-PCR, Western blotting, and siRNA knockdown.

**Results:**

We identified 255 senescence-related differentially expressed genes enriched in oxidative stress and inflammatory pathways. WGCNA and machine learning converged on 10 hub genes, with SIRT1 prioritized based on network centrality and diagnostic performance (AUC = 0.997, 95% CI: 0.992–1.000) and reduced expression in plaque samples. Virtual screening nominated Poliumoside as a high-affinity SIRT1 binder with stable binding dynamics in molecular simulations. In HUVECs, Poliumoside (40 μM) mitigated H_2_O_2_-induced injury and activated the SIRT1/NRF2/HO-1 axis; these effects were attenuated by SIRT1 knockdown.

**Conclusion:**

This study highlights SIRT1 as a senescence-related theranostic candidate in atherosclerosis and identifies Poliumoside as a natural SIRT1 activator with endothelial protective activity *in vitro*.

## Introduction

Atherosclerosis (AS), the primary underlying pathology of cardiovascular diseases (CVD), is responsible for approximately 31% of global deaths annually (Mensah et al., 2019). Despite significant advances in lipid-lowering therapies, particularly statins, a substantial residual risk of adverse cardiovascular events persists in many patients (Cholesterol Treatment Trialists et al., 2010; Grundy et al., 2019). This underscores that the pathogenesis of AS extends beyond lipid dyslipidemia, involving complex processes such as chronic inflammation, endothelial dysfunction, and oxidative stress (Libby et al., 2011; Gistera and Hansson, 2017).

Accumulating evidence now positions cellular senescence as a crucial driver in the initiation and progression of AS (Childs et al., 2016; Grootaert et al., 2018). Senescent cells accumulate in atherosclerotic plaques, where they promote plaque instability through the sustained secretion of pro-inflammatory cytokines and matrix-degrading enzymes. Studies have demonstrated that the clearance of senescent cells can attenuate atherosclerosis, highlighting the therapeutic potential of targeting this pathway (Roos et al., 2016). Furthermore, several established anti-aging interventions, including metformin, rapamycin, and NAD^+^ precursors, have exhibited cardiovascular benefits in both preclinical and clinical studies (Barzilai et al., 2016; Rajman et al., 2018; Sciarretta et al., 2018). These observations collectively suggest that aging and atherosclerosis share common molecular regulatory networks. Consequently, targeting genes at this interface represents a promising yet underexplored therapeutic strategy, and the identification of specific, senescence-related targets remains a critical unmet need in AS management.

In addition to soluble SASP mediators, senescent cells actively release senescence-associated extracellular vesicles, which have emerged as important vehicles for intercellular transmission of senescence signals (Krupa et al., 2026). These vesicles can carry proteins, lipids, DNA fragments, mRNAs, microRNAs, and other non-coding RNAs, thereby transferring pro-inflammatory, pro-oxidative, and senescence-promoting cues to neighboring or distant cells (Głuchowska et al., 2022). In the vascular microenvironment, senescence-associated extracellular vesicles may facilitate communication among endothelial cells, vascular smooth muscle cells, macrophages, and immune cells, amplifying SASP signaling and promoting bystander senescence, a process also referred to as SASP-mediated senescent drift. Such EV-mediated propagation of senescence may contribute to endothelial dysfunction, inflammatory cell recruitment, vascular remodeling, extracellular matrix degradation, and ultimately atherosclerotic plaque progression and instability (Okawa et al., 2024). Therefore, senescence in atherosclerosis should be considered not only as a cell-autonomous process but also as a multicellular communication network involving soluble SASP factors and extracellular vesicle-mediated signaling. This concept further supports the need to identify senescence-related diagnostic and therapeutic targets capable of modulating oxidative stress, inflammation, and intercellular senescence propagation (Smirnova et al., 2023).

Natural products present a compelling avenue for this challenge. Historically, they have been invaluable sources for pharmacologically active compounds, with approximately 50% of approved drugs originating from or inspired by natural compounds (Newman and Cragg, 2020). Their structural diversity, generally favorable safety profiles, and inherent multi-target capabilities offer distinct advantages for tackling complex diseases like AS (Harvey et al., 2015; Atanasov et al., 2021). In the cardiovascular disease field, numerous natural products, including resveratrol, berberine, and curcumin, have demonstrated atheroprotective effects through diverse mechanisms (Carrizzo et al., 2013; Feng et al., 2017; Feng et al., 2019). However, systematic screening of natural compounds against specific senescence-related therapeutic targets in AS remains limited. The integration of modern computational approaches, such as molecular docking, molecular dynamics simulations, and machine learning, now offers a powerful means to efficiently identify bioactive compounds from massive natural product libraries (Pinzi and Rastelli, 2019; Vamathevan et al., 2019).

To this end, we employed an integrative systems biology approach to discover senescence-related therapeutic targets and corresponding natural product interventions for AS. We analyzed transcriptomic data from the GSE21545 dataset and identified cellular senescence-related differentially expressed genes (CS-DEGs) by comparing carotid atherosclerotic plaque samples with matched peripheral blood mononuclear cells (PBMCs). Subsequent weighted gene co-expression network analysis (WGCNA) and dual machine learning algorithms (LASSO and SVM-RFE) prioritized SIRT1 as a core candidate hub gene showing strong dataset-level discrimination and biological relevance to oxidative stress regulation. We then conducted virtual screening of a large-scale natural product library using multi-stage molecular docking and molecular dynamics simulations, nominating Poliumoside as a candidate SIRT1 binder/modulator. Finally, *in vitro* experiments showed that Poliumoside activates the NRF2/HO-1 signaling axis in a SIRT1-dependent manner and mitigates H_2_O_2_-induced endothelial injury. Together, these findings prioritize SIRT1 as a senescence-related theranostic candidate in AS and support Poliumoside as a promising natural product lead for further preclinical evaluation.

## Materials and methods

### Data acquisition and preprocessing

The GSE21545 microarray dataset was obtained from the Gene Expression Omnibus (GEO) database (Barrett et al., 2013), utilizing the Affymetrix® GPL570 platform. This dataset encompasses 126 specimens from atherosclerotic plaques alongside 97 peripheral blood mononuclear cell (PBMC) specimens. Data processing was conducted through R software (version 4.2.0). The robust multi-array average (RMA) algorithm facilitated background adjustment and normalization procedures. The sva package’s ComBat function was employed to eliminate batch-related variations (Leek et al., 2012).

### Differential expression analysis

Gene expression differences between plaque and PBMC samples were assessed using the limma package. Differentially expressed genes (DEGs) were defined as those with |log_2_FC| > 1 and adjusted P < 0.05 (Benjamini–Hochberg FDR). Visual representations were generated through ggplot2 for volcano plots and pheatmap for heatmap illustrations.

### Integration with CellAge database

From the CellAge database (https://genomics.senescence.info/cells/), we extracted 867 genes associated with cellular senescence—a curated collection derived from gene manipulation studies across various human cell models. The overlap between plaque-enriched DEGs and CellAge entries was identified using the Venn package, yielding cellular senescence-related DEGs (CS-DEGs).

### Weighted gene Co-expression network analysis

Weighted gene co-expression network analysis (WGCNA) was performed using the WGCNA R package to construct a co-expression network for atherosclerotic plaque patients and controls. Sample data were preprocessed and outliers were removed. A correlation matrix was constructed using the WGCNA package. The optimal soft-thresholding power (β = 13) was selected to convert the correlation matrix to an adjacency matrix. A topological overlap matrix (TOM) was created based on the adjacency matrix. Using TOM-based dissimilarity measures, average linkage hierarchical clustering was employed to classify genes with similar expression patterns into gene modules. Module eigengenes were calculated and correlated with clinical traits. The blue module (532 genes) was selected based on positive correlation with plaque pathology (r = 0.67, P = 2 × 10^−30^). The intersection of blue module genes and CS-DEGs yielded 30 candidate genes. The current analytical framework is primarily focused on intracellular molecular discovery and validation, aiming to establish an interpretable and reproducible pipeline for target and compound prioritization.

### Machine learning-based feature selection

Two distinct machine learning methodologies were implemented for hub gene identification. LASSO (least absolute shrinkage and selection operator) regression employs regularization to enhance predictive accuracy through simultaneous variable selection and complexity adjustment during generalized linear model fitting. The optimal λ parameter was identified via 10-fold cross-validation using the glmnet R package, with response type configured as “binomial” and alpha set to “1”. SVM-RFE (support vector machine-recursive feature elimination) iteratively constructs models and identifies optimal features through sequential backward selection guided by SVM’s maximum margin principle [24]. Cross-validation during training yielded minimum-error values as feature genes. The e1071, kernlab, and caret packages facilitated SVM classifier implementation. Both algorithms independently screened hub genes, with their intersection—obtained via the Venn package—yielding 10 core hub genes as potential biomarkers. To prioritize hub genes, we applied two complementary machine learning methods: LASSO regression and SVM-RFE. LASSO performs variable selection and regularization simultaneously, effectively identifying genes with the strongest contribution to the outcome while controlling overfitting in high-dimensional transcriptomic data. SVM-RFE ranks features based on their impact on the SVM classification margin, capturing non-linear relationships and interaction effects that LASSO may not fully resolve. These two methods therefore operate on distinct statistical principles and offer complementary perspectives on feature importance. Taking the intersection of their outputs enhances the robustness and biological relevance of hub gene selection, as genes retained by both algorithms are more likely to represent true positive biomarkers with consistent predictive relevance across different modelling assumptions, reducing false positives that would arise from relying on a single method alone (Bao et al., 2023).

### Diagnostic performance evaluation

Differential expression of the 10 hub genes between atherosclerotic plaque patients and controls was visualized using the ggboxplot function with different colors representing the groups. The stat_compare_means function was added for statistical analysis of inter-group differences with significance levels marked by symbols. To evaluate diagnostic efficiency, the area under the curve (AUC) of receiver operating characteristic (ROC) curves was calculated for each hub gene. ROC curves were generated using the pROC package.

### Gene set enrichment analysis

Atherosclerotic patients were divided into high and low expression groups according to the median SIRT1 expression. Differential analysis was performed between high and low expression groups, followed by GSEA evaluation using GSEA software (version 4.3.2) with gene sets from the Molecular Signatures Database (MSigDB).

### Functional enrichment analysis

Gene Ontology (GO) was used to classify and annotate gene functions. Functional enrichment analysis was performed on three GO domains including biological process (BP), cellular component (CC), and molecular function (MF). Kyoto Encyclopedia of Genes and Genomes (KEGG) methodology was used to analyze metabolic pathways involving genes. Enrichment analysis was performed using the clusterProfiler R package (version 4.0) to determine biological functions and related pathways of the 10 hub genes (Yu et al., 2012). Statistical significance was set at adjusted P < 0.05.

### Immune cell infiltration analysis

The CIBERSORT algorithm was utilized to characterize immune profiles of atherosclerotic plaque patients versus controls. Infiltration patterns of 22 immune cell types were displayed through violin plots generated with the ggplot2 package, revealing differences between patient groups. Spearman rank correlation assessed relationships between SIRT1 gene expression and immune cell infiltration levels, with lollipop plots illustrating correlations between SIRT1 and distinct immune cell populations.

### Protein-protein interaction network analysis

The STRING database (version 11.5) facilitated protein-protein interaction (PPI) network construction with a confidence threshold exceeding 0.4. Networks incorporated the 10 hub genes alongside their interacting partners. Cytoscape software (version 3.9.1) enabled network visualization and topological analysis.

### Virtual screening and molecular docking

A total of 5,565 natural compounds from the MedChemExpress Natural Product Library (HY-L021P) were processed using LigPrep module in Schrödinger Suite 2024-2, generating approximately 13,000 3D conformers with ionization states at pH 7.4 ± 0.5. The crystal structure of SIRT1 (PDB ID: 4ZZH) was retrieved from the RCSB Protein Data Bank and prepared using Protein Preparation Wizard with hydrogen addition, bond order assignment, missing residue filling, and energy minimization using OPLS4 force field. Virtual screening employed a hierarchical docking protocol in Glide: high-throughput virtual screening (HTVS) with docking score cutoff ≤ −4 kcal/mol, standard precision (SP) docking cutoff ≤ −5 kcal/mol, and extra precision (XP) docking cutoff ≤ −6 kcal/mol (Friesner et al., 2006). Top-ranked compounds from XP docking underwent molecular mechanics generalized Born surface area (MM-GBSA) binding free energy calculations using Prime module. Compounds with ΔG_bind ≤ −30 kcal/mol were selected for molecular dynamics simulation.

### Molecular dynamics simulation

A 100-ns all-atom molecular dynamics (MD) simulation was performed using GROMACS 2022.3. Ligand topology was generated using AmberTools22 with General Amber Force Field (GAFF) and Restrained Electrostatic Potential (RESP) charges. Protein was parameterized using Amber99sb-ildn force field. The protein-ligand complex was solvated in TIP3P water box with a minimum distance of 10 Å from box edges and neutralized with Na^+^ ions.

Energy minimization was performed using steepest descent algorithm followed by conjugate gradient method until the maximum force was <1000 kJ/mol/nm. The system was equilibrated through NVT ensemble (100 ps at 300 K using V-rescale thermostat) and NPT ensemble (100 ps at 300 K and 1 bar using Parrinello-Rahman barostat). Production MD (100 ns with 2 fs time step) was conducted with V-rescale thermostat (300 K, τ = 0.1 ps) and Parrinello-Rahman barostat (1 bar, τ = 2.0 ps). Long-range electrostatics were calculated using Particle Mesh Ewald (PME) method with 10 Å cutoff for short-range interactions. LINCS algorithm was applied to constrain all bonds.

Trajectory analysis included root mean square deviation (RMSD), root mean square fluctuation (RMSF), hydrogen bonds, radius of gyration (Rg), and solvent-accessible surface area (SASA) using GROMACS analysis tools. Principal component analysis (PCA) was performed to construct the Gibbs free energy landscape. MM-GBSA calculations with per-residue energy decomposition were performed on the last 20 ns trajectory to identify key binding residues.

### Cell culture and treatments

Human umbilical vein endothelial cells (HUVECs) were sourced from Procell Life Science and Technology Co., Ltd. (Wuhan, China, catalog CL-0122) and maintained in Dulbecco’s Modified Eagle Medium (DMEM, 4.5 g/L glucose) supplemented with 10% fetal bovine serum (FBS), 100 U/mL penicillin, and 100 μg/mL streptomycin under standard conditions (37 °C, humidified atmosphere, 5% CO_2_). Passages 3-8 were utilized throughout experiments.

Poliumoside (HY-N0033, MedChemExpress) was reconstituted in dimethyl sulfoxide (DMSO) at 40 mM stock concentration. Hydrogen peroxide (H_2_O_2_, H112515, Aladdin) induced oxidative stress (300 μM for 48 h). For cytoprotection assays, cells received Poliumoside pretreatment (40 μM) for 24 h prior to H_2_O_2_ exposure. Final DMSO concentration remained at ≤0.1% to prevent solvent toxicity.

### Cell viability assay

Cell viability was assessed using Cell Counting Kit-8 (CCK-8) assay (Servicebio, Wuhan, China). HUVECs were seeded at a density of 2 × 10^3^ cells per well in 96-well plates and allowed to adhere overnight. For cytotoxicity testing, cells were treated with varying concentrations of Poliumoside (0–100 μM) for 24, 48, and 72 h. For cytoprotection testing, cells were pretreated with Poliumoside (40 μM) for 24 h, then exposed to varying concentrations of H_2_O_2_ (0–3000 μM) for 48 h. After treatments, 10 μL CCK-8 solution was added to each well and incubated for 1 h at 37 °C. Absorbance was measured at 450 nm using a microplate reader (BioTek). Cell viability was calculated as a percentage relative to untreated control cells.

### RNA extraction and quantitative real-time PCR

Total RNA was extracted from HUVECs using TRIzol reagent (Sigma-Aldrich) according to the manufacturer’s protocol. RNA concentration and purity were assessed using a NanoDrop spectrophotometer (Thermo Fisher Scientific). First-strand cDNA was synthesized from 1 μg total RNA using a reverse transcription kit from Accurate Biotechnology (Hunan) Co., Ltd (Changsha, China). Quantitative real-time PCR (qRT-PCR) was performed on a QuantStudio 5 Real-Time PCR System (Thermo Fisher Scientific) using a qPCR kit from Accurate Biotechnology (Hunan) Co., Ltd (Changsha, China). The thermal cycling program consisted of initial denaturation at 95 °C for 10 min, followed by 40 cycles of 95 °C for 15 s and 60 °C for 1 min. Melting curve analysis was performed to verify the specificity of amplification. Relative gene expression was calculated using the 2^(-ΔΔCt)^ method with β-actin as the internal control. Primer sequences for the 10 hub genes and β-actin are listed in Table 1.

**TABLE 1:** Primer sequences used for quantitative real-time PCR.

Gene	Forward primer (5′-3′)	Reverse primer (5′-3′)
SIRT1	GAG​TGG​CAA​AGG​GAG​CAG​A	TCT​GGC​ATG​TCC​CAC​TAT​C
β-actin	GAT​CAT​TGC​TCC​TCC​TGA​GC	ACT​CCT​GCT​TGC​TGA​TCC​AC
EWSR1	CCT​AGA​GGG​GAA​AGC​GAG​AG	TGG​GTG​GTC​TGT​GCA​TAT​C
PINK1	GCCTACATTGCCCCAGAA	GAGGAACCTGCCGAGATG
LMNA	GTGGATGAGGAGGGCAAG	CCGGTAAGTCAGCAAGGG
HDAC4	GCACATGAAGCCACCAG	CCCCAAGTCTGAACCCCA
MAGOHB	CTG​AAC​AAG​GGT​TGA​GGA​GAA​A	TTA​GAG​AAC​GGG​GAC​AAA​GA
DDAH2	GGA​CTC​CCT​TCT​CCA​CCA​A	TTC​TTG​TTT​CTT​CAC​CTG​TCT​CC
KDM4C	AGC​TCG​ATT​TTC​CAC​AGC​CT	AAA​CCT​GGA​GCT​CAG​CAC​TC
ING5	TCC​AGA​ACG​CCT​ACA​GCA​AG	TGC​CCT​CCA​TCT​TGT​CCT​TC
LRRK2	CCT​GGA​GGA​TCT​GCT​GGT​GTT	AAT​TTG​CAC​AGA​AGT​GAC​CAA​CC

### Western blot analysis

Cells were lysed in ice-cold RIPA lysis buffer (FD009, FDbio) supplemented with protease inhibitor cocktail and phosphatase inhibitors (PhosSTOP, Roche). Lysates were centrifuged at 12,000 rpm for 15 min at 4 °C, and supernatants were collected. Protein concentrations were determined using BCA Protein Assay Kit (P0010S, Beyotime). Equal amounts of protein (20–40 μg) were separated by 10% or 12% SDS-polyacrylamide gel electrophoresis (SDS-PAGE) gel kits (NCM Biotech) and transferred to polyvinylidene difluoride (PVDF) membranes (Millipore). Membranes were blocked with 5% non-fat milk in Tris-buffered saline containing 0.1% Tween-20 (TBST) for 1 h at room temperature, then incubated overnight at 4 °C with primary antibodies: anti-SIRT1 (1:1000, F0526, Selleck), anti-NRF2 (1:1000, ab76026, Abcam), anti-HO-1 (1:1000, 81281-1-RR, Proteintech), and anti-β-actin (1:5,000, RM 2001, Ray Antibody Biotech). After washing three times with TBST, membranes were incubated with horseradish peroxidase (HRP)-conjugated secondary antibodies (1:5,000, RM3003, Ray Antibody Biotech) for 1 h at room temperature. Protein bands were visualized using enhanced chemiluminescence (ECL) substrate (FD8020, FDbio) on a UVP GelStudio PLUS imaging system (Analytik Jena) and quantified using ImageJ software (NIH). Protein expression levels were normalized to β-actin.

### Gene knockdown by small interfering RNA (siRNA)

SIRT1 knockdown was performed using small interfering RNA (siRNA) transfection with Lipofectamine 2000 (Thermo Fisher Scientific) according to the manufacturer’s protocol. Three siRNA sequences targeting different regions of human SIRT1 were designed and synthesized by Sangon Biotech (Shanghai, China): siRNA1 (hSIRT1-712): sense strand 5′-AGA​UAU​UAA​UAC​AAU​UGA​A-3′, antisense strand 5′-UUC​AAU​UGU​AUU​AAU​AUC​U-3'; siRNA2 (hSIRT1-880): sense strand 5′-AGC​GAU​GUU​UGA​UAU​UGA​A-3′, antisense strand 5′-UUC​AAU​AUC​AAA​CUC​GCU-3'; siRNA3 (hSIRT1-940): sense strand 5′-GGA​AAU​AUA​UCC​UGG​ACA​A-3′, antisense strand 5′-UUG​UCC​AGG​AUA​UAU​UUC​C-3'. Scrambled non-targeting siRNA was used as negative control (siNC). HUVECs were seeded in 6-well plates at 50%–60% confluence and transfected with 50 nM siRNA. Knockdown efficiency was confirmed at 48–72 h post-transfection by qRT-PCR and Western blot analysis. The siRNA with highest knockdown efficiency was selected for subsequent experiments.

### Statistical analysis

Statistical analyses were performed using R software (version 4.2.0) and GraphPad Prism (version 8.0). Data are presented as mean ± standard deviation (SD) from at least three independent experiments performed in triplicate. Normal distribution of data was assessed using Shapiro-Wilk test. For two-group comparisons, Student’s t-test (for normally distributed data) or Wilcoxon rank-sum test (for non-normally distributed data) was used. Multiple group comparisons were performed using one-way analysis of variance (ANOVA) followed by Tukey’s *post hoc* test. Spearman correlation coefficient was used to assess relationships between variables. A two-tailed P value <0.05 was considered statistically significant (*P < 0.05, **P < 0.01, ***P < 0.001, ****P < 0.0001).

## Results

### Target identification workflow

**FIGURE 1:**
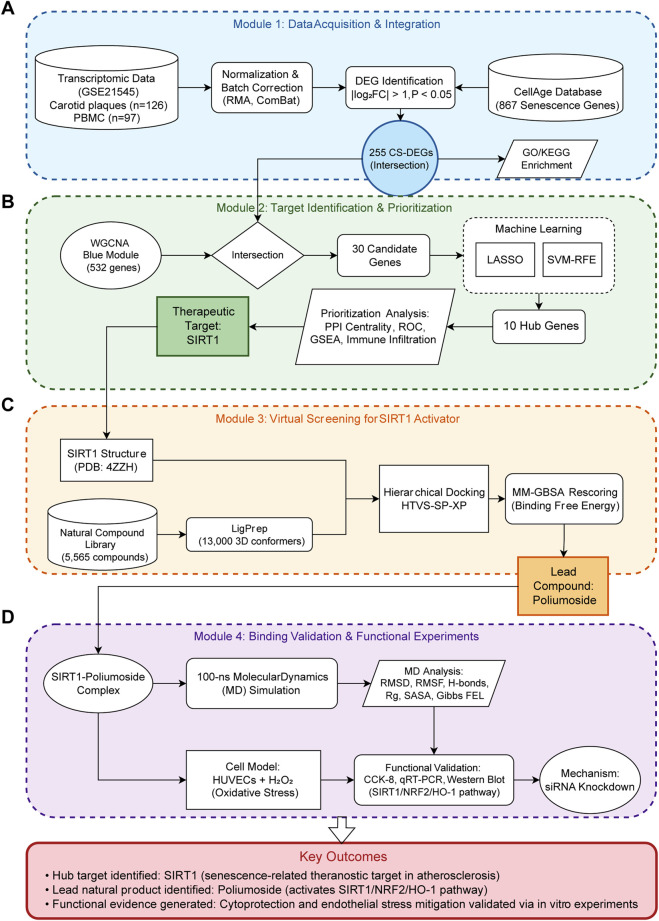
Overview of the integrative systems-biology workflow. The pipeline consists of four modules: **(A)** Data Acquisition and Integration; **(B)** Target Identification and Prioritization; **(C)** Virtual Screening for SIRT1 Activators; **(D)** Binding Validation and Functional Experiments.

In this study, we established an integrative systems-biology framework to identify a therapeutic target for atherosclerosis and to discover a natural activator with experimental validation. As illustrated in Figure 1, the overall workflow comprises four consecutive modules (A) data acquisition and integration, encompassing transcriptomic dataset retrieval, differential expression analysis, and intersection with senescence-associated gene resources (B) target identification and prioritization, involving WGCNA, machine-learning feature selection (LASSO and SVM-RFE), ROC analysis, immune infiltration, and PPI network construction, which ultimately pinpointed SIRT1 as the core candidate (C) virtual screening for SIRT1 activators, including hierarchical docking of a natural product library, MM-GBSA rescoring, and molecular dynamics simulations; and (D) binding validation and functional experiments, comprising cellular viability assays, qRT-PCR, Western blotting, and siRNA-mediated knockdown in H_2_O_2_-injured HUVECs (Figure 1).

### Identification of cellular senescence-related differentially expressed genes in atherosclerosis

**FIGURE 2:**
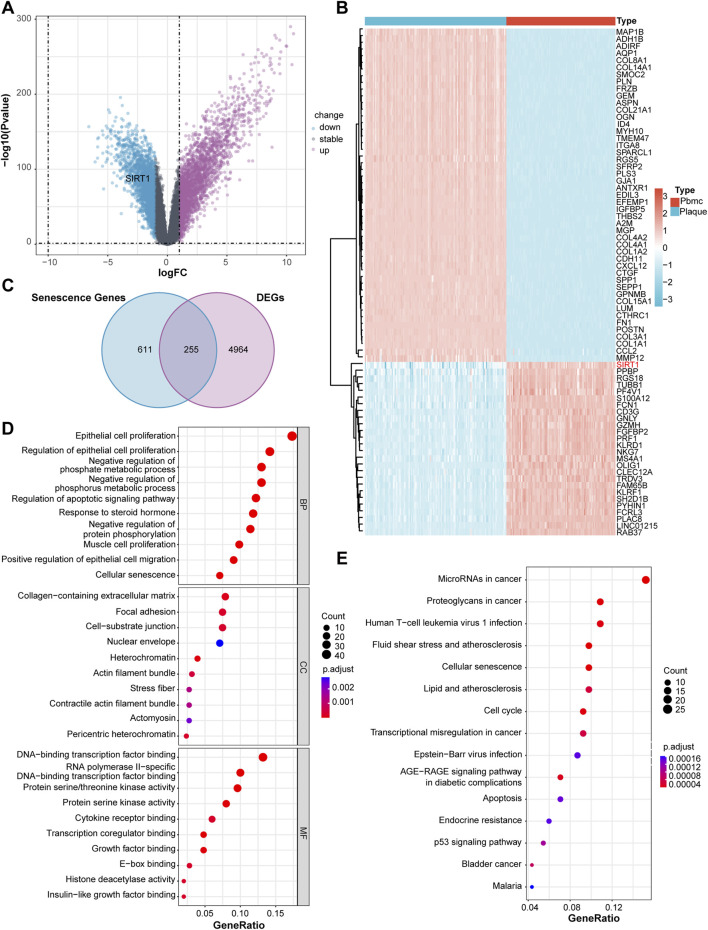
Identification of cellular senescence-related genes in atherosclerotic plaques. **(A)** Volcano plot of 5,219 DEGs. Red: upregulated (n = 2,509); blue: downregulated (n = 2,710); gray: non-significant. **(B)** Heatmap of DEG expression patterns. **(C)** Venn diagram showing overlap between 5,219 DEGs and 867 senescence genes, identifying 255 CS-DEGs. **(D)** GO enrichment of 255 CS-DEGs. **(E)** KEGG pathway enrichment analysis of 255 CS-DEGs.

To identify genes specifically enriched in atherosclerotic lesions, we analyzed the GSE21545 dataset comprising 126 carotid atherosclerotic plaque samples and 97 matched peripheral blood mononuclear cell (PBMC) samples from patients undergoing carotid endarterectomy. Using the limma package with stringent criteria (|log_2_FC| > 1 and adjusted P < 0.05), we identified 5,219 differentially expressed genes (DEGs), including 2,509 upregulated and 2,710 downregulated genes in plaques compared to PBMCs (Figures 2A,B). Hierarchical clustering analysis revealed distinct expression patterns between plaque and PBMC samples, confirming tissue-specific gene expression signatures (Figure 2B).

Growing evidence suggests that aging-related processes share common molecular mechanisms with atherosclerosis. Several drugs used for age-related disease management, such as metformin and aspirin, have demonstrated anti-aging properties in preclinical and clinical studies. To explore whether atherosclerosis and aging are governed by overlapping genetic networks, we intersected the 5,219 plaque-specific DEGs with 867 cellular senescence-associated genes from the CellAge database. This analysis identified 255 cellular senescence-related DEGs (CS-DEGs) that are specifically dysregulated in atherosclerotic plaques (Figure 2C) (Tyrrell and Goldstein, 2021).

To characterize the functional implications of these CS-DEGs, we performed comprehensive enrichment analyses. Gene Ontology (GO) enrichment analysis revealed that these 255 CS-DEGs were significantly enriched in biological processes related to cellular response to oxidative stress, regulation of apoptotic process, inflammatory response, and DNA damage response (Figure 2D). Kyoto Encyclopedia of Genes and Genomes (KEGG) pathway analysis showed significant enrichment in canonical aging-associated pathways, including p53 signaling pathway, cellular senescence, AGE-RAGE signaling pathway, and senescence-associated secretory phenotype (SASP)-related inflammatory cytokine production (Figure 2E). These findings indicate the critical involvement of senescence-related mechanisms in atherosclerosis pathogenesis and provide a molecular foundation for targeting cellular senescence as a therapeutic strategy (Zhao et al., 2025).

### WGCNA identifies disease-relevant gene modules

**FIGURE 3:**
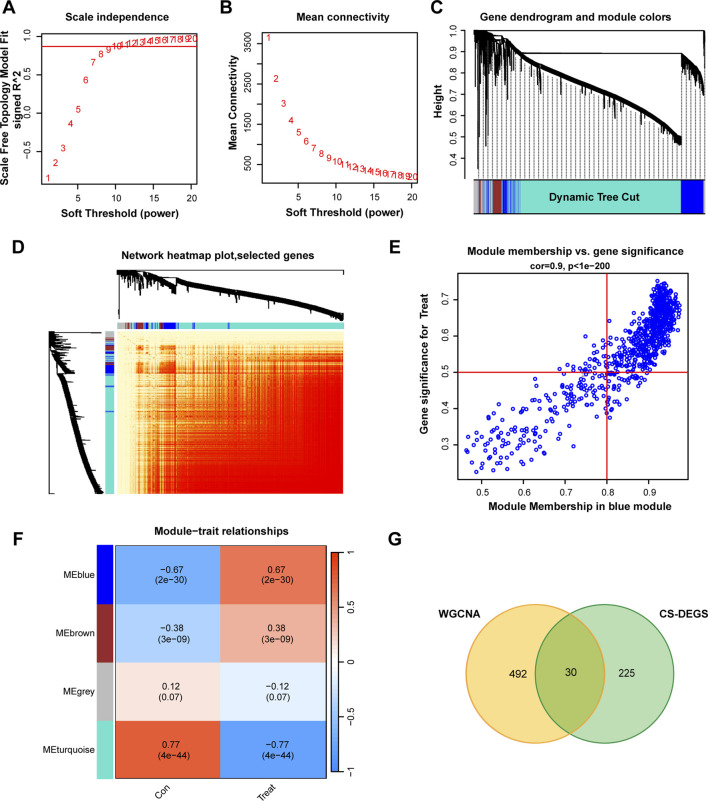
WGCNA identifies disease-relevant gene modules. **(A)** Scale independence analysis. β = 13 selected (R^2^ > 0.85). **(B)** Mean connectivity analysis. **(C)** Hierarchical clustering dendrogram showing 12 modules. **(D)** TOM heatmap of 12 modules. **(E)** MM vs. GS scatter plot for blue module (r = 0.9, P < 1 × 10^−200^). **(F)** Module-trait correlation heatmap. Blue module: r = 0.67, P = 2 × 10^−30^. **(G)** Venn diagram: 255 CS-DEGs ∩ 532 blue module genes = 30 candidates.

We performed weighted gene co-expression network analysis (WGCNA) on the normalized expression matrix of the GSE21545 dataset (plaque and PBMC samples) to construct a scale-free co-expression network and identify disease-associated gene modules. A soft-thresholding power of β = 13 was selected to achieve a scale-free topology fit index (R^2^ > 0.85) (Figures 3A,B). Dynamic tree cutting identified 12 distinct modules, with sizes ranging from 52 to 1,834 genes (Figure 3C). The blue module (532 genes) showed the strongest positive correlation with the plaque phenotype (r = 0.67, P = 2 × 10^−30^) (Figure 3F). To focus on senescence-related signals, we intersected the blue-module genes with the 255 cellular senescence-related DEGs (CS-DEGs), yielding 30 candidate genes for downstream machine-learning selection (Figure 3G).

### Machine learning prioritizes SIRT1 as a core hub gene with strong dataset-level discrimination

**FIGURE 4:**
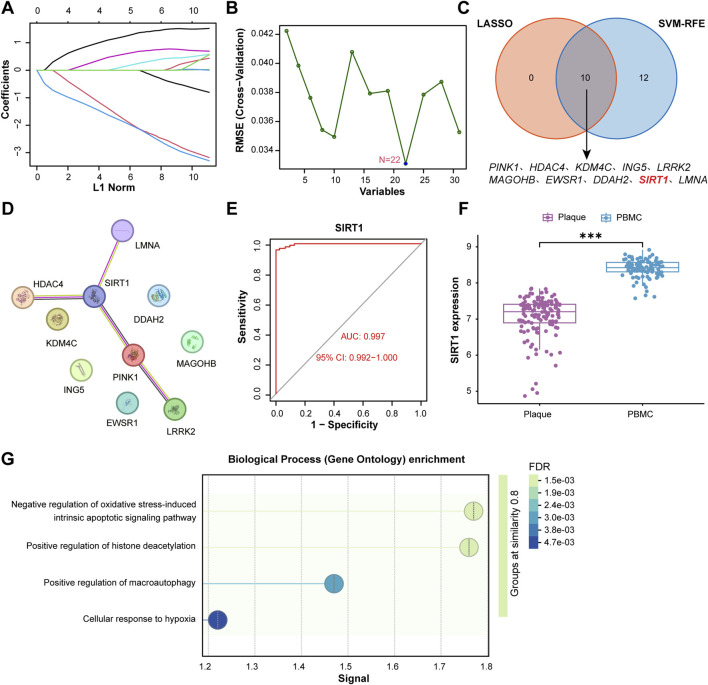
Machine learning identifies SIRT1 as a key biomarker. **(A)** LASSO coefficient profiles. Ten genes retained at optimal λ. **(B)** SVM-RFE feature selection. Twenty-two genes selected. **(C)** Venn diagram: LASSO (10 genes) ∩ SVM-RFE (22 genes) = 10 core genes. **(D)** PPI network of 10 hub genes. **(E)** ROC curve of SIRT1 (AUC = 0.997, 95% CI: 0.992–1.000). **(F)** SIRT1 expression in plaques vs. PBMCs (***P < 0.001). **(G)** GO enrichment of SIRT1-associated genes.

We applied two complementary machine learning algorithms to identify the most critical biomarkers from the 30 candidate genes. LASSO regression with 10-fold cross-validation identified 10 genes with non-zero coefficients at the optimal lambda value (Figure 4A). In parallel, support vector machine-recursive feature elimination (SVM-RFE) analysis identified 22 genes as the optimal feature subset based on classification accuracy (Figure 4B). The intersection of genes identified by both algorithms yielded 10 core hub genes: SIRT1, PINK1, HDAC4, KDM4C, ING5, LRRK2, MAGOHB, EWSR1, DDAH2, and LMNA (Figure 4C).

Expression profiling confirmed that all 10 core genes were significantly differentially expressed between atherosclerotic plaques and PBMCs (Figures 4D–F). Among these genes, we prioritized SIRT1 for in-depth characterization based on several lines of evidence. First, protein-protein interaction network analysis revealed that SIRT1 occupies a central hub position, exhibiting extensive interactions with multiple hub genes including PINK1 (Figure 4D). Second, SIRT1 is widely recognized as a longevity gene with established protective effects in age-related cardiovascular disorders. Third, functional enrichment analysis of SIRT1-associated genes highlighted the negative regulation of oxidative stress-induced apoptotic signaling (Figure 4G), a key protective mechanism against cellular senescence (Imai and Guarente, 2016; Dai et al., 2018).

Receiver operating characteristic (ROC) curve analysis showed that SIRT1 discriminated plaque samples from PBMCs with an AUC of 0.997 (95% CI: 0.992–1.000, P < 0.001). While this strong separation is consistent with marked transcriptomic differences between the two sample types, it should be interpreted cautiously in terms of clinical generalizability. Collectively, these results support SIRT1 as a prioritized candidate for downstream mechanistic interrogation and natural product-based modulation.

### Functional characterization of SIRT1 reveals its central regulatory role in atherosclerosis

**FIGURE 5:**
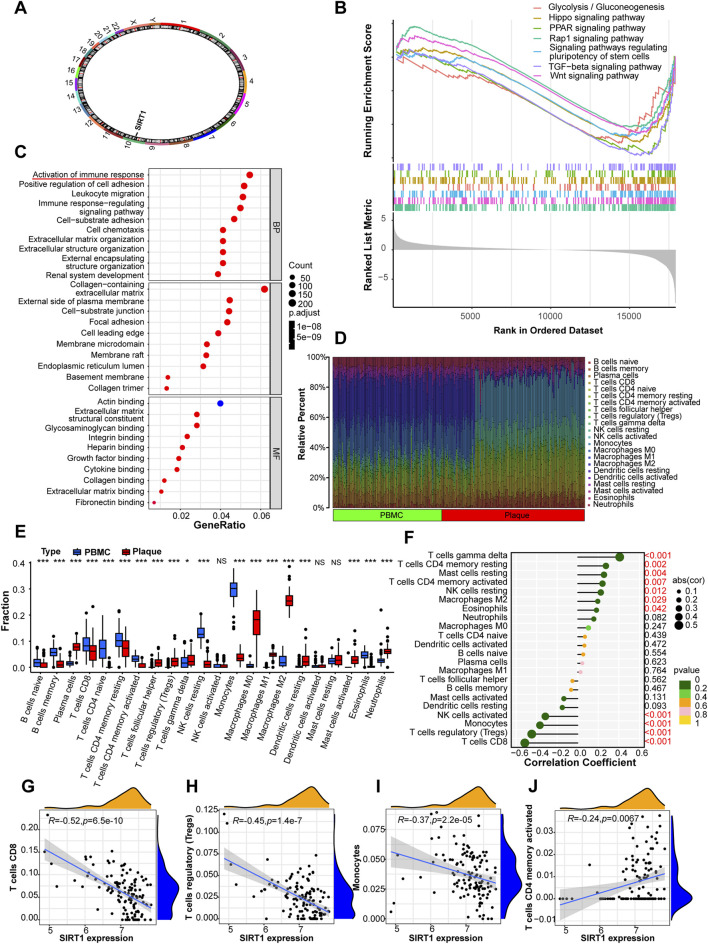
SIRT1-associated immune landscape in atherosclerotic plaques. **(A)** Chromosomal location of SIRT1 (10q21.3). **(B)** GSEA showing pathways downregulated in SIRT1-low group. **(C)** GO enrichment highlighting leukocyte migration (P = 4.49 × 10^−31^), cell-substrate adhesion (P = 1.26 × 10^−28^), and cell chemotaxis (P = 5.12 × 10^−26^). **(D)** Correlation heatmap of 22 immune cell types. **(E)** Box plots comparing immune cell infiltration between plaques (n = 126) and PBMCs (n = 97). **(F)** Lollipop plot of SIRT1 correlation with 22 immune cell types. **(G–J)** Scatter plots: **(G)** CD8^+^ T cells (R = −0.52, P = 6.5 × 10^−10^), **(H)** Tregs (R = −0.45, P = 1.4 × 10^−7^), **(I)** monocytes, **(J)** activated CD4^+^ memory T cells (R = 0.24, P = 0.0067).

Having identified SIRT1 as the most promising biomarker, we sought to comprehensively characterize its functional roles and immunological context in atherosclerotic plaques. SIRT1 (Sirtuin 1), located on chromosome 10q21.3 (Figure 5A), is a NAD^+^-dependent deacetylase widely recognized as a “longevity gene” due to its established roles in extending lifespan and delaying aging-related pathologies across multiple organisms. Notably, SIRT1 has been extensively implicated in the pathogenesis of age-related diseases including cardiovascular disease, neurodegenerative disorders, and metabolic syndrome. Its selection as our primary target was further supported by its central position in the protein-protein interaction network (Figure 4D), where it functions as a hub regulator coordinating multiple cellular processes. Moreover, SIRT1 serves as an upstream regulator of PINK1 (PTEN-induced kinase 1), a key mediator of mitophagy and cellular quality control, thereby positioning SIRT1 at a critical node governing both cellular senescence and metabolic homeostasis (Dai et al., 2018).

To elucidate the biological processes modulated by SIRT1 in atherosclerosis, we performed gene set enrichment analysis (GSEA) by stratifying patients into SIRT1-high and SIRT1-low expression groups based on median expression levels. GSEA revealed striking differences in pathway activation between the two groups (Figure 5B, Extended Data Figure 4). In SIRT1-high patients, we observed significant enrichment of pathways associated with adaptive immune activation, including B cell receptor signaling pathway, T cell receptor signaling pathway, Th1 and Th2 cell differentiation, Th17 cell differentiation, as well as cell cycle and DNA replication pathways (Extended Data Figure 4B). These findings suggest that elevated SIRT1 expression is associated with enhanced immune surveillance and cellular proliferation capacity. Conversely, SIRT1-low patients exhibited downregulation of critical metabolic and signaling pathways, including glycolysis/gluconeogenesis, Hippo signaling pathway, PPAR signaling pathway, Rap1 signaling pathway, pathways regulating pluripotency of stem cells, TGF-beta signaling pathway, and Wnt signaling pathway (Figure 5B). This metabolic and signaling dysfunction in SIRT1-low plaques aligns with the concept of cellular senescence, characterized by mitochondrial dysfunction, metabolic reprogramming, and impaired stress responses.

Gene Ontology enrichment analysis of SIRT1-associated genes further revealed prominent enrichment in immune-related biological processes, particularly leukocyte migration (P = 4.49 × 10^−31^), cell-substrate adhesion (P = 1.26 × 10^−28^), and cell chemotaxis (P = 5.12 × 10^−26^) (Figure 5C). These findings underscore the intimate connection between SIRT1 expression and immune cell recruitment and positioning within atherosclerotic plaques, suggesting that SIRT1 may regulate plaque composition and stability through modulation of immune infiltration.

To systematically evaluate the relationship between SIRT1 expression and immune microenvironment in atherosclerotic plaques, we employed CIBERSORT algorithm to estimate the relative abundance of 22 immune cell subsets in plaque and PBMC samples. Comparative analysis revealed significant differences in immune cell composition between plaques and PBMCs (Figures 5D,E), with plaques showing enrichment of monocytes, macrophages, and activated immune cells, consistent with their inflammatory nature. Correlation analysis between SIRT1 expression and immune cell infiltration levels identified several significant associations (Figure 5F). Notably, SIRT1 expression showed strong negative correlations with CD8+ T cells (R = −0.52, P = 6.5 × 10−10, Figure 5G) and regulatory T cells (Tregs, R = −0.45, P = 1.4 × 10−7, Figure 5H), both of which are associated with chronic inflammation and tissue damage in atherosclerosis. A correlation with monocytes was also observed (Figure 5I). In contrast, SIRT1 displayed a positive correlation with activated CD4^+^ memory T cells (R = 0.24, P = 0.0067, Figure 5J), which may contribute to adaptive immune regulation. These findings were further corroborated by additional immune cell correlations, including positive associations with γδ T cells (R = 0.41, P = 2.1 × 10^−6^), resting CD4^+^ memory T cells (R = 0.27, P = 0.0019), and M2 macrophages (R = 0.19, P = 0.029), alongside negative correlations with activated NK cells (R = −0.32, P = 0.00032) (Extended Data Figures 3A–F). Collectively, these data indicate that SIRT1 expression is associated with distinct immune cell composition signatures in this dataset; however, because the comparison involves plaque tissue versus PBMCs, immune-infiltration estimates should be interpreted cautiously with respect to disease-specific immune microenvironment inference and warrant validation using tissue-matched designs.

Together, our functional characterization reveals that SIRT1 downregulation in atherosclerotic plaques is accompanied by metabolic dysfunction, impaired signaling pathway activation, and a pro-inflammatory immune landscape. These findings position SIRT1 as a master regulator integrating cellular senescence, metabolic homeostasis, and immune modulation in atherosclerosis, and provide strong rationale for targeting SIRT1 as a therapeutic strategy to restore plaque stability and promote vascular health.

### Virtual screening nominates poliumoside as a candidate SIRT1 modulator

**FIGURE 6:**
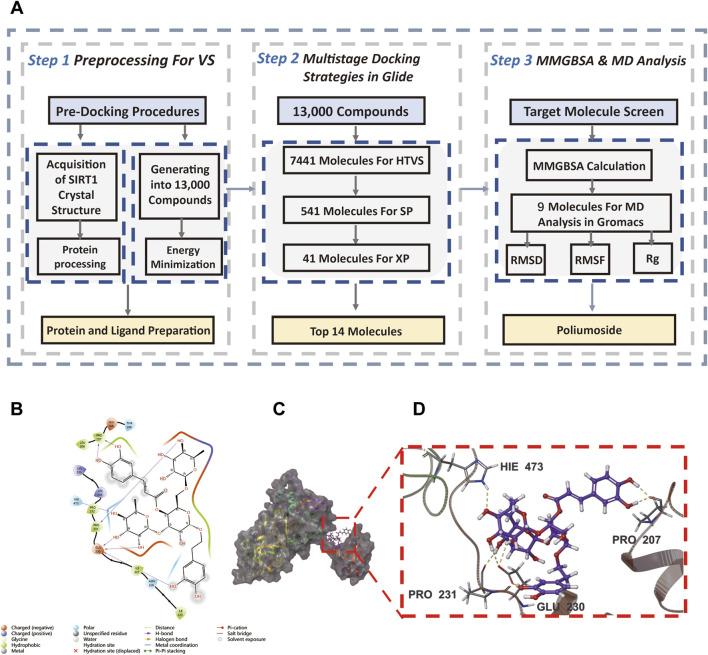
Molecular docking nominates Poliumoside as a candidate SIRT1 binder/modulator. **(A)** Virtual screening workflow. Sequential filtering: HTVS → SP → XP → MM-GBSA yielded 9 candidates. Poliumoside: XP = −8.645 kcal/mol, MM-GBSA = −34.15 kcal/mol. **(B)** 2D interaction diagram. **(C)** 3D structure showing Poliumoside in SIRT1 active site. **(D)** Detailed binding interactions with key residues.

Having prioritized SIRT1 as a senescence-related candidate theranostic node in this transcriptomic setting, we next sought to identify natural product-derived compounds that may modulate SIRT1. To discover natural product-derived SIRT1 activators, we performed systematic virtual screening of 5,565 natural compounds from the MedChemExpress Natural Product Library (HY-L021P). The three-dimensional crystal structure of SIRT1 (PDB ID: 4ZZH) was retrieved from the RCSB Protein Data Bank and prepared using Schrödinger Suite. Ligand structures were processed using LigPrep to generate approximately 13,000 3D conformers with proper ionization states at physiological pH. We employed a hierarchical virtual screening strategy with progressively increasing precision to efficiently identify high-affinity SIRT1 binders (Figure 6A). The multi-stage protocol consisted of: (1) high-throughput virtual screening (HTVS) of 13,000 conformers, retaining compounds with docking scores ≤ −4 kcal/mol; (2) standard precision (SP) docking, yielding compounds with scores ≤ −5 kcal/mol; (3) extra precision (XP) docking with a stringent threshold of ≤ −6 kcal/mol; and (4) molecular mechanics generalized Born surface area (MM-GBSA) binding free energy calculations, selecting compounds with ΔG_bind ≤ −30 kcal/mol. This multistage protocol ultimately yielded 9 candidate compounds that satisfied all filtering criteria.

Among the 9 candidates, Poliumoside, a phenylpropanoid glycoside, exhibited the most favorable binding profile with an XP docking score of −8.645 kcal/mol and MM-GBSA ΔG_bind of −34.15 kcal/mol, surpassing all other candidates. Structural analysis revealed that Poliumoside forms extensive interactions with key residues in the SIRT1 active site, including multiple hydrogen bonds and hydrophobic contacts (Figure 6B). Three-dimensional visualization demonstrated that Poliumoside occupies the SIRT1 active site with excellent shape complementarity, engaging critical residues surrounding the NAD^+^ cofactor binding site (Figures 6C,D). Based on its superior binding affinity and extensive molecular interactions, Poliumoside was selected as the lead compound for subsequent molecular dynamics simulation and experimental validation.

### Molecular dynamics simulation confirms binding stability of SIRT1-Poliumoside complex

**FIGURE 7:**
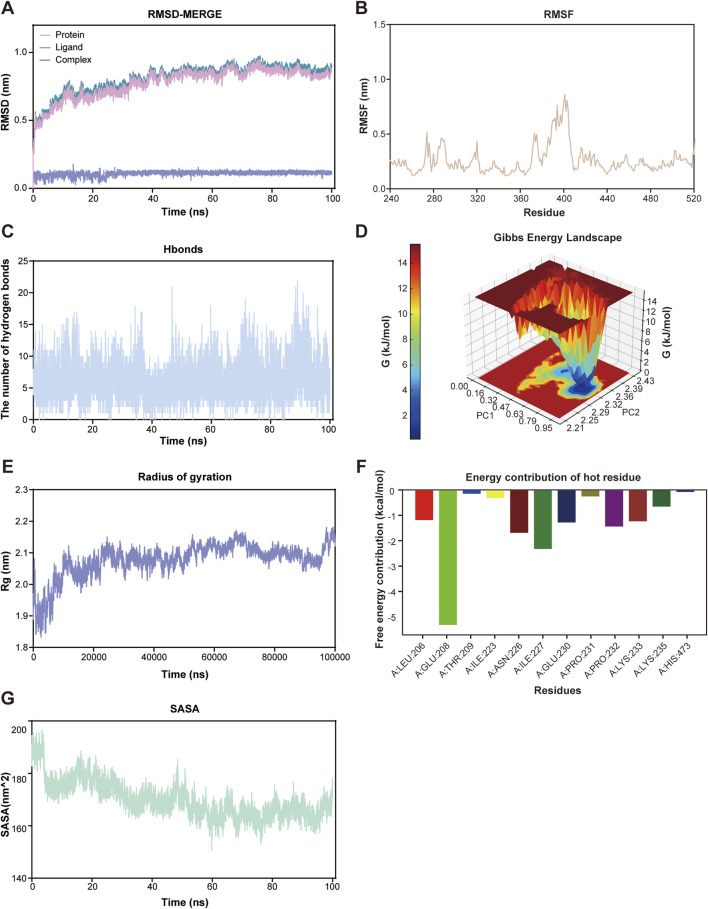
Molecular dynamics simulation validates binding stability. **(A)** RMSD during 100 ns simulation. Equilibrium after ∼20 ns. Protein: ∼0.2–0.3 nm; ligand: ∼0.15–0.25 nm. **(B)** RMSF. Binding site: <0.15 nm; loops: >0.3 nm. **(C)** Hydrogen bonds: average 7 (range 5–9). **(D)** Gibbs energy landscape showing stable conformational states. **(E)** Radius of gyration: ∼2.35 nm. **(F)** MM-GBSA decomposition: key residues with ΔG < −2 kcal/mol. **(G)** SASA: ∼150–160 nm^2^.

To further validate the stability and reliability of the SIRT1-Poliumoside interaction predicted by molecular docking, we performed a 100-nanosecond all-atom molecular dynamics (MD) simulation using GROMACS 2022.3 under physiological conditions. Root mean square deviation (RMSD) analysis revealed that the SIRT1-Poliumoside complex reached equilibrium after approximately 20 ns, after which the system remained stable throughout the simulation (Figure 7A). The protein backbone RMSD stabilized at ∼0.2–0.3 nm, ligand RMSD at ∼0.15–0.25 nm, and the complex RMSD showed consistent values, indicating that Poliumoside maintained stable binding without significant conformational drift. Root mean square fluctuation (RMSF) analysis demonstrated that residues in the ligand-binding pocket exhibited lower fluctuations (<0.15 nm), while regions distant from the binding site showed higher flexibility as expected (Figure 7B).

Hydrogen bond analysis revealed an average of 7 hydrogen bonds (range: 5–9) between Poliumoside and SIRT1 throughout the simulation (Figure 7C), demonstrating sustained interactions. Gibbs free energy landscape analysis, constructed based on the first two principal components, revealed distinct low-energy basins (Figure 7D), indicating that the complex predominantly occupies stable conformational states. Additional analyses showed that the radius of gyration remained constant at ∼2.35 nm (Figure 7E) and solvent-accessible surface area remained stable at ∼150–160 nm^2^ (Figure 7G), confirming sustained protein stability and ligand burial within the binding pocket. MM-GBSA energy decomposition identified key residues contributing most favorably to binding (ΔG < −2 kcal/mol) (Figure 7F). Collectively, these results support a stable SIRT1-Poliumoside binding mode and prioritize Poliumoside as a candidate SIRT1 modulator for subsequent experimental evaluation.

### Poliumoside activates the SIRT1/NRF2/HO-1 pathway and exerts cytoprotective effects in endothelial cells

**FIGURE 8:**
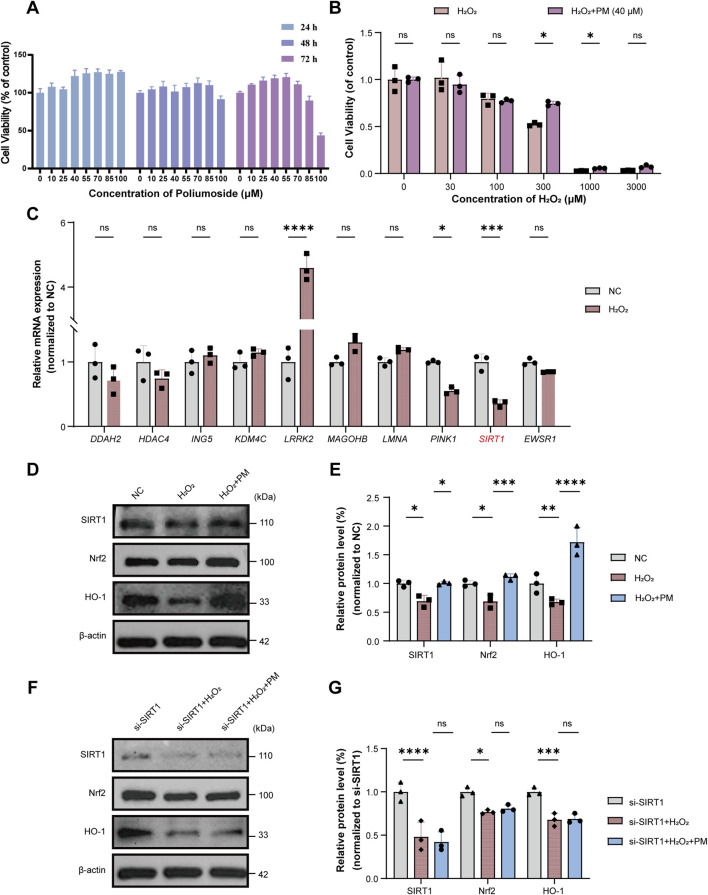
Poliumoside protects endothelial cells via SIRT1/NRF2/HO-1 pathway. **(A)** CCK-8 assay showing cell viability of HUVECs treated with Poliumoside (0–100 μM) for 24, 48, and 72 h. **(B)** CCK-8 assay showing cell viability of HUVECs exposed to H_2_O_2_ (0–3000 μM) with or without 40 μM Poliumoside pretreatment for 24 h. **(C)** qPCR analysis of mRNA expression levels of 10 hub genes in HUVECs treated with 300 μM H_2_O_2_ for 48 h versus control group. **(D)** Western blot analysis of SIRT1, NRF2, and HO-1 protein expression in control group, H_2_O_2_-treated group (300 μM, 48 h), and H_2_O_2_ + Poliumoside group. **(E)** Quantitative analysis of protein expression levels corresponding to **(D)**. **(F)** Western blot analysis of SIRT1, NRF2, and HO-1 protein expression in SIRT1-knockdown HUVECs across control group, H_2_O_2_-treated group, and H_2_O_2_ + Poliumoside group. **(G)** Quantitative analysis of protein expression levels corresponding to **(F)**. Data are presented as mean ± SD, n = 3. Ns, not significant; *p < 0.05, **p < 0.01, ***p < 0.001, ****p < 0.0001 (B, C unpaired two-tailed Student’s t-test; E, G one-way ANOVA with Tukey’s *post hoc* test).

Having supported a stable SIRT1–Poliumoside binding mode through computational modeling, we next sought to experimentally examine its endothelial protective activity and pathway engagement *in vitro*. We performed comprehensive *in vitro* studies using human umbilical vein endothelial cells (HUVECs). CCK-8 assays revealed that Poliumoside exhibited no significant cytotoxicity at concentrations up to 40 μM across 24, 48, and 72 h of treatment (Figure 8A), establishing a safe working concentration range. We then assessed cell viability using CCK-8 assays with H_2_O_2_ concentrations ranging from 0 to 3000 μM, both with and without 40 μM Poliumoside pretreatment for 24 h. The results demonstrated that treatment of HUVEC cells with 300 μM H_2_O_2_ for 48 h reduced cell viability to 53%, approaching the half-maximal inhibitory concentration (IC50). Pretreatment with 40 μM Poliumoside for 24 h significantly increased cell viability to 75%, effectively protecting cells from oxidative stress-induced damage. These conditions were therefore established as optimal for subsequent validation experiments (Figure 8B).

QPCR analysis of the 10 core hub genes in HUVECs treated with 300 μM H_2_O_2_ for 48 h versus control group revealed that SIRT1 and its downstream target PINK1 exhibited consistent downregulation in response to oxidative stress (Figure 8C), validating their critical roles in cellular response to oxidative injury and confirming our bioinformatic predictions.

Western blot analysis revealed that H_2_O_2_ treatment (300 μM, 48 h) significantly suppressed SIRT1, NRF2, and heme oxygenase-1 (HO-1) protein levels. Remarkably, Poliumoside pretreatment (40 μM for 24 h) not only prevented H_2_O_2_-induced SIRT1 downregulation but also significantly upregulated NRF2 and HO-1 expression, demonstrating activation of the SIRT1/NRF2/HO-1 antioxidant signaling axis (Figures 8D,E). To confirm that Poliumoside exerts its antioxidant effects through SIRT1 activation, we performed SIRT1 knockdown experiments using small interfering RNA (siRNA). SIRT1 knockdown completely abolished Poliumoside-induced upregulation of NRF2 and HO-1 (Figures 8F,G), demonstrating that SIRT1 is indispensable for Poliumoside’s antioxidant effects. These results establish that Poliumoside exerts potent cytoprotective effects through SIRT1-dependent activation of the NRF2/HO-1 antioxidant pathway, providing experimental validation for its therapeutic potential in atherosclerosis.

## Discussion

This study presents an integrative workflow linking systems-level target prioritization with natural product screening and *in vitro* validation in an endothelial injury model. Transcriptomic analyses identified 255 cellular senescence-related DEGs enriched in oxidative stress and inflammatory pathways. WGCNA and dual machine learning approaches converged on SIRT1 as a prioritized hub gene with strong plaque-versus-PBMC discrimination in this dataset and plausible biological relevance to stress resistance. Structure-based virtual screening and molecular dynamics simulations nominated Poliumoside as a promising SIRT1-binding lead. Consistently, in HUVECs exposed to H_2_O_2_, Poliumoside attenuated cellular injury and activated the SIRT1/NRF2/HO-1 axis in a SIRT1-dependent manner. Together, our findings support SIRT1 as a senescence-related theranostic candidate in atherosclerosis and highlight Poliumoside as a natural product lead for further preclinical evaluation.

Cellular senescence is recognized as a core mechanism driving multiple age-related diseases, including atherosclerosis (Lopez-Otin et al., 2013; Childs et al., 2015). Our study innovatively combined senescence biology with cardiovascular disease research to reveal significant dysregulation of 255 cellular senescence-related genes in atherosclerotic plaques. The significance of this discovery lies in providing a molecular basis for explaining why drugs traditionally used for metabolic diseases, such as metformin, rapamycin, and aspirin, exhibit anti-aging effects and cardiovascular protection (Johnson et al., 2013; Barzilai et al., 2016). These drugs share the common feature of modulating senescence-related pathways, including AMPK-mTOR signaling, oxidative stress response, and chronic inflammation. Our functional enrichment analysis showed that these CS-DEGs were significantly enriched in the p53 signaling pathway, DNA damage response, and inflammatory pathways, which highly align with the core pathological mechanisms of atherosclerosis—chronic inflammation and endothelial dysfunction. This finding strongly supports the concept of “senescence as a therapeutic target for atherosclerosis” and provides theoretical foundations for therapeutic strategies. Particularly noteworthy is that senescence-targeted therapeutic strategies (senotherapy), including senolytic and senomorphic agents, have shown potential for improving cardiovascular function in animal models in recent years (Roos et al., 2016; Jia et al., 2019). Our study provides preclinical molecular evidence for this therapeutic direction.

It should be noted that the *in vitro* experiments in this study were primarily designed for early-stage pharmacological screening rather than full recapitulation of cellular senescence. Hydrogen peroxide (H_2_O_2_)-induced oxidative stress was selected as the experimental model due to its well-established role in endothelial dysfunction and the induction of senescence-associated phenotypes in the context of atherosclerosis (Jimenez Trinidad et al., 2021; Berenjabad et al., 2022). This model provides a relevant “senescence-associated injury background” for screening potential SIRT1 modulators, but it does not capture the full spectrum of cellular senescence, such as long-term phenotypic changes or broader molecular markers. Future studies should incorporate more comprehensive senescence characterization, including multi-marker panels and long-term functional assays, to fully validate the anti-senescence effects of candidate compounds.

SIRT1 (Silent Information Regulator 1), a member of the NAD^+^-dependent deacetylase family, is termed a “longevity gene” due to its critical roles in lifespan extension, metabolic regulation, and stress resistance (Howitz et al., 2003). Our study provides compelling evidence for SIRT1’s central role in atherosclerosis through multi-level bioinformatics analysis. WGCNA analysis identified a blue module containing 532 genes that was highly correlated with plaque pathology (r = 0.67, P = 2 × 10^−30^) and significantly enriched in cellular senescence and oxidative stress pathways (Stein and Matter, 2011; Gorenne et al., 2013). Through intersection with CS-DEGs and dual machine learning screening, SIRT1 emerged as the most critical biomarker. Remarkably, SIRT1 showed strong classification performance in this data for distinguishing plaque tissue from PBMC samples. Given the cross-tissue nature of this comparison, this metric likely reflects both disease-related changes and tissue-of-origin signatures, and therefore should be interpreted as an internal dataset discrimination result rather than definitive evidence of clinical diagnostic utility.

The progression of atherosclerosis is accompanied by complex immune cell infiltration and inflammatory responses (Wolf and Ley, 2019). Our immune infiltration analysis revealed that SIRT1 expression was significantly correlated with multiple immune cell subsets, suggesting that SIRT1 may maintain vascular homeostasis by modulating immune responses. The negative correlation with CD8^+^ T cells suggests that SIRT1 may suppress cytotoxic T cell-mediated vascular damage, while positive correlations with M2 macrophages and resting NK cells indicate promotion of anti-inflammatory and tissue repair phenotypes. This immune profile indicates that SIRT1 may not only protect endothelial cells through intrinsic cellular mechanisms but also delay plaque progression by shaping an anti-inflammatory immune microenvironment. These findings align with recent evidence demonstrating immunomodulatory roles of sirtuins in cardiovascular diseases.

Natural products occupy a unique position in drug discovery due to their chemical diversity, multi-target activity, and relatively favorable safety profiles. Compared to synthetic SIRT1 activators such as SRT1720, natural products typically exhibit broader biological activities and better tolerability. For instance, resveratrol, the most famous SIRT1 activator, has shown cardiovascular protective effects in numerous preclinical studies, but its clinical application is limited by low bioavailability and rapid metabolism. We successfully identified Poliumoside as a candidate drug with excellent SIRT1 binding properties from 5,565 natural compounds using a hierarchical virtual screening strategy. Poliumoside is a phenylethanoid glycoside extracted from Stachys sieboldii (Lamiaceae family), traditionally used for anti-inflammatory and antioxidant purposes, but its role in cardiovascular protection has been rarely reported. Molecular dynamics simulations and *in vitro* assays together support Poliumoside as a promising lead with stable binding behavior *in silico* and acceptable cellular tolerance under the tested conditions, warranting further pharmacokinetic, pharmacodynamic, and safety evaluations in future studies.

Our mechanistic studies revealed that Poliumoside exerts endothelial protective effects through activating the SIRT1/NRF2/HO-1 signaling axis. Western blot analysis showed that Poliumoside pretreatment significantly upregulated protein expression levels of SIRT1, NRF2, and HO-1 in H_2_O_2_-induced oxidative stress models. NRF2 (Nuclear factor erythroid 2-related factor 2) is the master regulator of cellular antioxidant responses, and SIRT1 enhances NRF2 stability and transcriptional activity through deacetylation modification. HO-1 (Heme oxygenase-1), as a downstream effector of NRF2, catalyzes heme degradation to produce biliverdin and carbon monoxide with antioxidant, anti-inflammatory, and anti-apoptotic effects. The SIRT1/NRF2/HO-1 pathway has been well-established as a critical defense mechanism against oxidative stress in cardiovascular diseases. Additionally, we observed upregulation of PINK1, a key regulator of mitophagy, suggesting that Poliumoside may also maintain cellular energy metabolic homeostasis by promoting clearance of damaged mitochondria. These findings provide mechanistic insights into the multi-faceted protective effects of Poliumoside.

Our findings suggest potential translational relevance. SIRT1 emerges as a candidate theranostic node that links senescence-related biology with oxidative stress regulation in atherosclerosis. The observed classification performance (AUC = 0.997) indicates that SIRT1 may help distinguish plaque tissue from PBMCs in this dataset; however, the clinical utility of SIRT1 for early detection, risk stratification, or blood-based monitoring will require validation in independent cohorts with appropriate vascular controls and dedicated blood-based sampling designs.

Moreover, SIRT1 not only functions as an intracellular deacetylase regulating transcriptional programs, but its activity may also influence extracellular vesicle (EV) biogenesis and secretion through the modulation of membrane trafficking and endosomal sorting complexes required for transport (ESCRT). Recent studies indicate that SIRT1 can regulate multivesicular body (MVB) formation and EV release via deacetylation of Rab GTPases and other membrane-associated proteins. In the context of our study, pharmacological activation of SIRT1 by poliumoside may, in addition to enhancing intracellular antioxidant and metabolic signaling, alter EV secretion and cargo composition. These EVs could serve as vehicles to transmit anti-senescence and antioxidant signals to neighboring vascular cells, thereby modulating the local vascular microenvironment (Smirnova et al., 2023). Mechanistically, SIRT1 may facilitate MVB trafficking to the plasma membrane through Rab27a/Rab11 deacetylation and selectively enrich antioxidant and SASP-related factors in EVs via ESCRT-dependent cargo sorting. Such EV-mediated intercellular communication could potentially reduce vascular inflammation, improve endothelial function, and mitigate the progression of atherosclerosis (Lee et al., 2019). Future studies could validate these mechanisms by assessing EV marker proteins (e.g., CD63, CD81), profiling senescence- or SASP-associated EV cargo, and combining SIRT1 modulation to determine causal relationships between SIRT1 activity, EV biology, and endothelial protection (Ding et al., 2023).

Poliumoside, as a candidate SIRT1-modulating natural product, may represent a complementary mechanistic avenue to existing atherosclerosis therapies by enhancing endogenous antioxidant defenses and endothelial resilience. Nevertheless, any potential add-on benefit to lipid-lowering therapy (e.g., statins) remains speculative and should be evaluated in appropriately designed *in vivo* and clinical studies. It is important to acknowledge the translational considerations associated with Poliumoside as a natural product candidate. As a polyphenolic glycoside, Poliumoside possesses a structurally complex scaffold with multiple hydroxyl groups and a glycosidic moiety, which may present challenges for membrane permeability, oral bioavailability, and metabolic stability-well-recognized limitations in natural product-based drug discovery. In terms of safety, existing studies employing Poliumoside in long-term rodent models of metabolic disease have not reported overt toxicity, providing preliminary evidence for its tolerability and supporting its classification as a low-toxicity natural compound (Xu et al., 2024; Gao et al., 2025; Williamson, 2025). Nevertheless, the effective concentration observed in our *in vitro* experiments (40 μM) underscores the need for dedicated pharmacokinetic and toxicological evaluation before any translational advancement. We therefore propose that Poliumoside is most appropriately positioned at this stage as a mechanistically informed starting scaffold rather than a terminal drug candidate. Its validated SIRT1-activating chemotype provides a rational basis for structure-activity relationship (SAR)-guided analog development, selective modification of the glycosidic linkage to improve bioavailability, and formulation optimization strategies such as nanoparticle encapsulation or prodrug design (Wiciński et al., 2023; Guo et al., 2024). These directions represent feasible and scientifically grounded paths toward translational development (Yang et al., 2020).

## Limitations

This study has several limitations. First, the primary transcriptomic analysis relied on GSE21545, which compares carotid plaque tissue with patient-matched PBMCs rather than healthy arterial tissue; therefore, part of the observed differential expression and module–trait associations may reflect tissue-of-origin signatures in addition to disease-related changes. Future studies should validate SIRT1 expression patterns and its potential diagnostic utility in independent cohorts that include appropriate vascular controls and alternative platforms. Second, the therapeutic potential of poliumoside was evaluated only *in vitro*; *in vivo* efficacy, pharmacokinetics, bioavailability, and systemic safety remain to be established before translational conclusions can be drawn. Third, computational screening and molecular dynamics simulations are inherently sensitive to protein conformation, force fields, and scoring functions; thus, predicted binding modes and relative affinities should be interpreted as prioritization evidence rather than definitive proof of target engagement. Fourth, while our data support a SIRT1-dependent activation of the NRF2/HO-1 axis in endothelial protection, deeper mechanistic resolution (e.g., NRF2 transcriptional activity, oxidative stress readouts, and senescence phenotypes) will further strengthen the causal chain. Fifth, this study did not experimentally assess EV secretion or cargo composition following SIRT1 activation, and the specific protein and RNA cargo within EVs associated with cellular senescence or SASP were not systematically analyzed. Consequently, the potential EV-mediated effects of poliumoside on the vascular microenvironment and their contribution to endothelial protection remain unclear. Finally, causal links between SIRT1-mediated EV changes and functional outcomes in endothelial cells or vascular tissue were not directly established. Future research integrating EV marker analysis, cargo profiling, and SIRT1 activation or inhibition experiments will be essential to extend the mechanistic framework from intracellular regulation to intercellular communication, providing a more comprehensive understanding of SIRT1’s role in endothelial aging and atherosclerosis.

## Conclusion

In summary, we present an integrative pipeline that links systems-level target prioritization with experimental follow-up to connect senescence-associated signals in atherosclerosis to a tractable therapeutic hypothesis. Our analyses prioritize SIRT1 as a candidate theranostic node associated with plaque-related transcriptional patterns and oxidative stress regulation. We further nominate Poliumoside as a natural product lead that shows favorable *in silico* binding behavior and SIRT1-dependent endothelial protective effects *in vitro*. Our findings provide mechanistic insights into the senescence-inflammation-oxidative stress axis in atherosclerosis and offer a foundational framework for developing innovative cardiovascular therapies. This work exemplifies a translational theranostic strategy, and future investigation into Poliumoside, from animal models to clinical trials, promises to advance senescence-targeting interventions for cardiovascular disease.

## Data Availability

The transcriptomic data analyzed in this study are publicly available in the Gene Expression Omnibus (GEO) repository under accession number GSE21545. The remaining data generated in this study are included in the article/Supplementary material. Further inquiries can be directed to the corresponding authors.
